# The medial clearspace is a risk factor for secondary dislocation following cast immobilization after closed reduction in closed ankle fracture dislocations

**DOI:** 10.1007/s00068-025-02803-z

**Published:** 2025-04-03

**Authors:** Verena Hecht, Eléonore Sophie Mosimann, Fabian Krause, Christophe Kurze, Thomas Lustenberger, Helen Anwander

**Affiliations:** Department of Orthopaedic Surgery and Traumatology, Inselspital, Bern University Hospital, University of Bern, Bern, Switzerland

**Keywords:** Ankle fracture dislocation, Medial clear space, Closed reduction, Cast immobilization, External fixation, Orthopedic trauma

## Abstract

**Purpose:**

Ankle fractures represent about 10% of all adult fractures, with increasing incidence. Dislocated ankle fractures often require delayed open reduction and internal fixation due to swelling, necessitating temporary stabilization using a cast or an external fixator. This study aims to assess risk factors for insufficient preliminary reduction immobilized by a cast, focusing on medial clearspace and posterior malleolus fragment size, to identify fractures that would benefit from initial stabilization with an external fixator.

**Methods:**

Patients treated for dislocated ankle fractures at our level-1 trauma center from 2011 to 2023 were retrospectively reviewed. The primary outcome was the rate of insufficient reduction during immobilization in a cast. Secondary outcomes included time to definitive surgery, length of surgery and hospital stay.

**Results:**

134 patients met the inclusion criteria. The most common fracture type was AO 44B3, with 71.6%. Sufficient reduction was achieved in 53.7% of patients. Multiple regression analyses revealed the initial medial clearspace at the time of dislocation as an independent risk factor for insufficient reduction after reduction. ROC-analysis revealed that a initial medial clearspace at the time of dislocation of 9 mm is a predictor for insufficient reduction with a sensitivity of 88% and a specificity of 55%.

**Conclusion:**

Initial medial clearspace was an important predictor for insufficient reduction in a cast, with 9 mm being identified as the cutoff for critical initial medial clearspace. Therefore, we recommend primary external fixation or acute internal fixation, if the soft tissue allows it for those patients with initial medial clearspace of > 9 mm. This approach may prevent secondary dislocation, reduce swelling, and expedite definitive surgery.

## Introduction

**Fig. 1:**
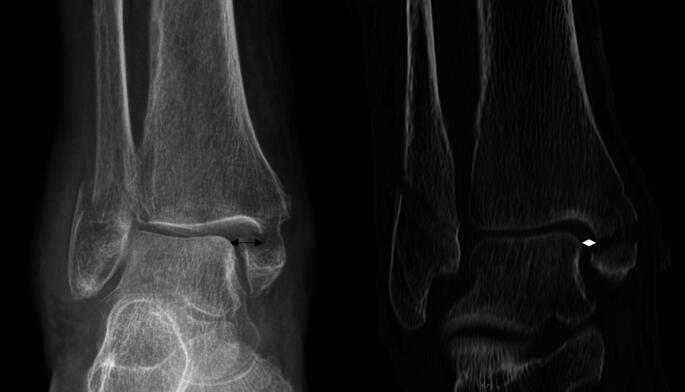
Anterior posterior radiograph of a trimalleolar ankle fracture. The left image shows the ankle in its prereduction state. The black arrow shows the medial clearspace expanded to 8.3 mm. The right image shows the ankle post reduction in computed tomography. The white diamond shows the measured medial clearspace of 4.2 mm

Ankle fractures account for 10% of all fractures, with incidence rising due to aging, obesity, and sports [1–3]. The Lauge-Hansen classification systems aid diagnosis, with supination-external rotation fractures being the most common [4], and a medial clear space more than 5 mm indicating syndesmosis or deltoid ligament injuries [9–12]. (Fig. 1)

Ankle joint dislocation is defined by misalignment of distal tibia and talus. While most dislocated ankle fractures require open reduction and internal fixation, acute swelling may necessitate temporary reduction, immobilization, and delayed definitive surgery. Two proven methods are described in the literature: immobilization in a cast or in an external fixator [13,–15]. 

Ankle fracture-dislocations are prone to reduction loss following closed reduction and casting. Recent literature demonstrated that 78.4% can be successfully reduced in a cast on the first attempt, but up to 43-50% require a second reduction after redislocation [16–18]. Redislocation increases pain, soft tissue complications, osteochondral lesions, and delays definitive fixation [18–20]. As the edema subsides and swelling decreases, the cast’s fit is reduced further risking reduction loss [5]. 

An external fixator, on the other hand, achieves reduction and stable fixation through axial traction and ligamentotaxis, but carries the risk of pin tract infection and requires surgery [21]. 

Given the limited evidence for a cast, this study aims to evaluate risk factors for insufficient preliminary reduction of ankle fractures immobilized in a cast.

## Patients and methods

### Patient selection

Patients treated in our level-1 trauma center for dislocated ankle fractures between January 2011 and April 2023 were retrospectively identified using the clinical electronic information system.

Inclusion criteria were the presence of an ankle fracture dislocation and age ≥ 18 years. Exclusion criteria were an open fracture, incomplete radiological documentation, definitive osteosynthesis within 24 h after trauma, history of fracture or surgery of the same ankle, a pathological fracture, refused informed consent to participate in a study or the immediate application of an external fixator. The indication to perform an immediate ankle spanning external fixator application was at the discretion of the attending surgeon.

The primary outcome was the rate of insufficient reduction during initial immobilization in the cast. Secondary outcomes included time to definitive surgery, length of surgery and hospital length of stay.

### Demographics and outcomes

All electronic records of the eligible patients were reviewed and the following data was abstracted and analyzed: demographics (age, gender, Body Mass Index (BMI)), comorbidities (diabetes mellitus, smoking, alcohol abuse, osteoporosis, vascular disease), fracture classification according to the AO/OTA [6–8], medial clearspace, presence and size of a posterior malleolar fragment (PMF), initial percentage of tibiotalar dislocation, treatment history (primary external fixation, primary cast immobilization, quality of reduction, time to surgery, duration of surgery and time to discharge).

### Definitions and measurements

An **ankle fracture dislocation** was defined as a loss of congruity of the ankle joint space, depicted by an enlargement of the medial clear space > 5 mm in the anterior posterior (ap) x-ray of the ankle and/or anteroposterior dislocation (subluxation) of the talus relative to the tibia of > 5 mm in the lateral x-ray of the ankle that was initially performed (Fig. 1) [22]. The medial clearspace was measured as the widest distance between the medial border of the talus and the lateral border of the medial malleolus [23]. If these criteria were met, immediate closed reduction of the ankle joint was performed under sedation, if possible due to pain, or under short anesthesia by the emergency doctor on call. The congruence of the ankle joint was therefore restored under xray control and a split plaster cast was applied.

The **initial dislocation** in **percentage** was measured using the x-ray. For this purpose the axis of the tibia and the talus were defined in the a anterior posterior radiograph and the dislocation of this two lines was measured [24]. In addition, the calculated dislocation was set in relation to the width of the talus at the level of the joint surface.

**Insufficient reduction** in the cast was defined by an incongruent tibiotalar joint with a widening of the medial clearspace > 5 mm on the anterior posterior view and/or an anterior-posterior dislocation of > 5 mm on the lateral view in the x-ray or the CT scan that was immediately performed after the initial reduction and casting [25]. 

The **sagittal diameter of the PMF** was measured in the CT scan. The longest extension in the sagittal plane was used for this purpose [26]. 

**Fig. 2:**
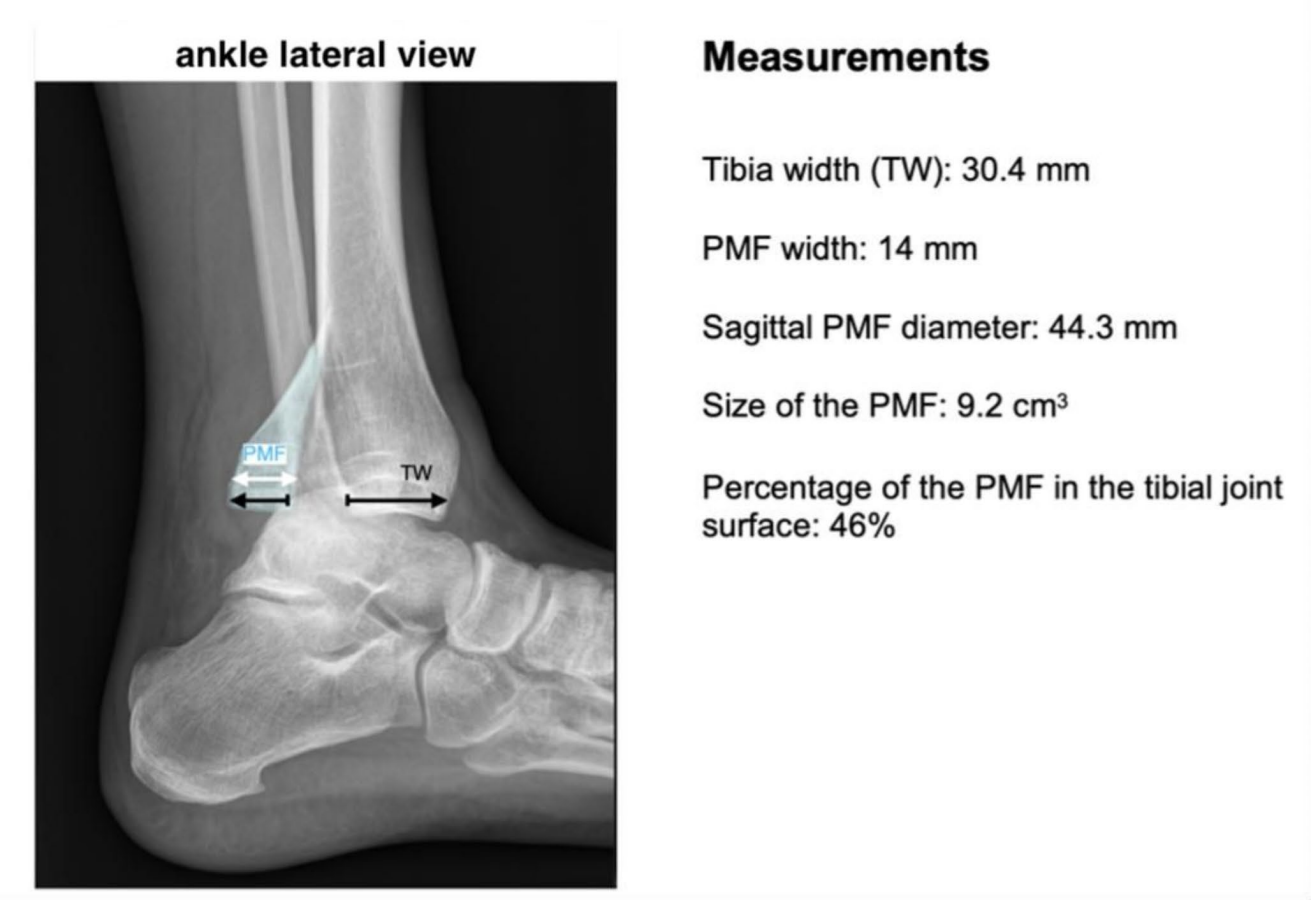
Lateral radiograph of a trimalleolar ankle fracture. The ratio between the width of the PMF and the width of the tibia defines the percentage of the PMF in the tibial joint surface, as well as the sagittal diameter

In contrast, the **percentage of the PMF in the tibial joint surface** was calculated using the lateral radiograph according to Fig. 2 [27]. 

The common classification according to Haraguchi was also used to classify posterior malleolar fractures, which divides the fractures into 3 types depending on extension and localization [28]. 

### Treatment

In our clinic, as standard of care, immediate closed fracture reduction under fluoroscopy is performed in the emergency department. Subsequently, a below-the-knee open circular cast is applied to retain the reduction. Following reduction and casting, computed tomography of the upper ankle joint is performed for exact fracture evaluation and preoperative planning.

After a decrease of the swelling, definitive open reduction and internal fixation is performed. Following surgery, the ankle is immobilized in a plantigrade position in a cast, with no weight bearing initially and partial weight bearing with crutches over time, for a total of 6–12 weeks depending on the fracture severity and soft tissue injuries. Low-molecular-weight heparin is routinely administered for thromboembolism prophylaxis. Routine follow-ups for clinical and radiologic evaluation are set at 3, 6, 12, 24, and 52 weeks.

### Statistical analysis

The patients were stratified into two groups: sufficient versus insufficient closed reduction. The p values for categorical variables were derived from the Pearson’s Chi-sqare or the 2-sided Fisher’s exact test. For continuous variables the Student’s t-test or the Mann-Whitney tests were deployed.

To identify risk factors independently associated with an insufficient reduction, a stepwise logistic regression model was utilized and risk factors from the bivariate analysis with a p value < 0.2 were included into the model.

Receiver operating characteristic (ROC) curves were constructed to analyze different variable’s discriminating power for predicting insufficient reduction, and the areas under the ROC curve were compared.

Values are reported as mean ± standard deviation (SD) for continuous variables and as percentages for categorical variables.

Differences were considered statistically significant when *p* ≤ 0.05. The data were analyzed using the Statistical Package for Social Sciences (SPSS Windows©), version 26 (SPSS Inc., Chicago, IL).

#### Ethical approval

for this study was received by the local ethical committee (2021 − 00646).

## Results

**Fig. 3:**
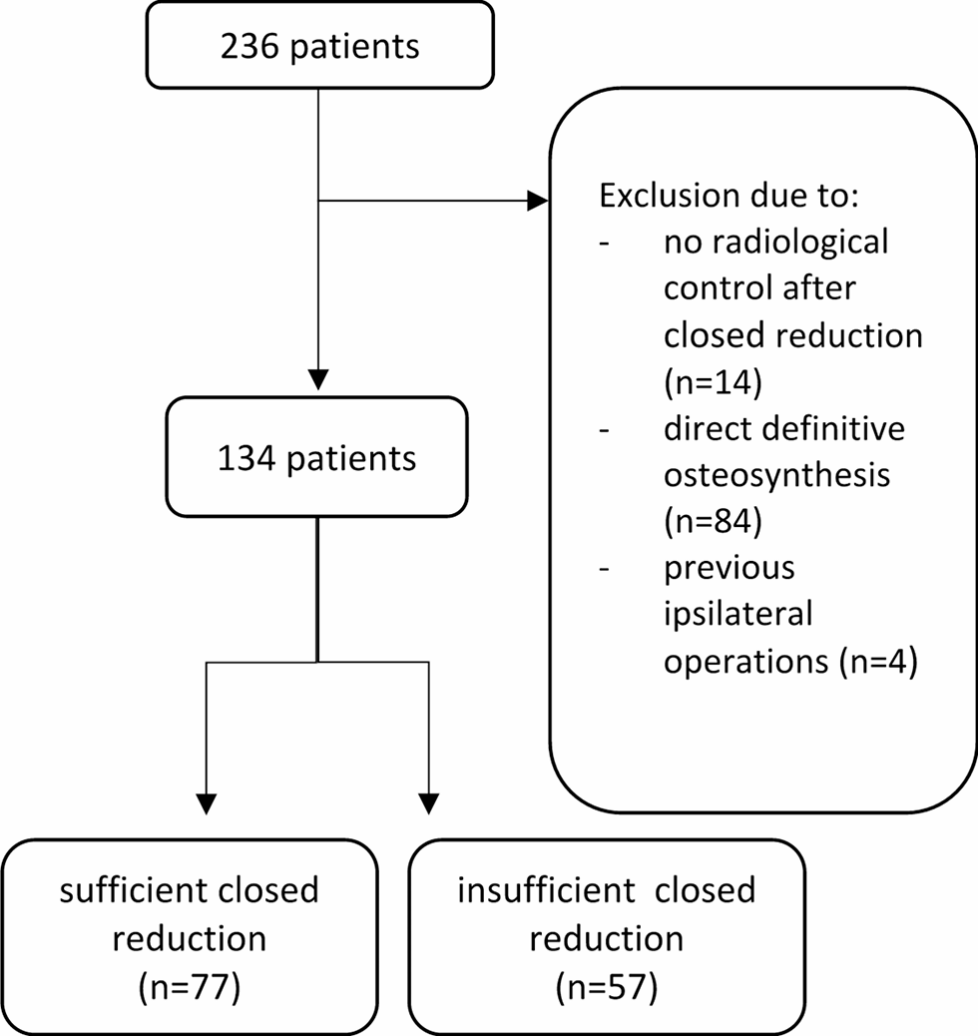
Flowchart

Between January 2011 und April 2023 a consecutive series of 236 adult patients with 236 dislocated ankle fractures were treated at our institution. A total of 102 cases were excluded, resulting in a study cohort of 134 patients with 134 dislocated ankle fractures (Fig. 3).

The study cohort included 77 women and 57 men with a mean age of 50.9 ± 17.5 years (range 18 to 88 years). The most common fracture type according to the AO classification was type 44B3 with 71.6% of the cases.

Of the 134 dislocated ankle fractures, in 77 patients (57.5%) a sufficient reduction in a cast was achieved, whereas 57 patients (42.5%) showed an insufficient reduction in a cast.

Demographic factors and comorbidities were comparable between patients with sufficient and insufficient ankle reduction (Table 1). No statistically significant differences were found comparing age, gender, BMI, smoking and alcohol abuse and comorbidities between the two groups.

**Table 1:** Comparison of the two groups regarding the demographic characteristics and comorbidities

	All patients (*n* = 134)	Sufficient reduction (*n* = 77)	Insufficient reduction (*n* = 57)	*p*
Age, years, mean±SD	50.9±17.5	50.9±17.9	50.9±17.0	0.976
Male/female, N (%)	57/77 (42.5/57.5)	33/44 (42.9/57.1)	24/33 (42.1/57.9)	0.536
BMI, kg/m², mean±SD	27.5±5.4	26.95±1.18	28.17±1.12	0.303
Smoker, N (%)	15 (11.2)	10 (9.7)	5 (12.9)	0.582
Alcohol abuse, N (%)	3 (2.2)	2 (2.6)	1 (1.8)	0.612
Hypertension, N (%)	11 (8.2)	6 (7.8)	5 (8.8)	1.000
Diabetes mellitus, N (%)	8 (6)	2 (2.6)	6 (10.5)	0.072
Osteoporosis, N (%)	10 (7.5)	5 (6.5)	5 (8.8)	0.743
Peripheral arterial disease, N (%)	1 (0.7)	0 (0)	1 (1.8)	0.425
Coronary heart disease, N (%)	6 (4.5)	3 (3.9)	3 (5.3)	0.699

Statistically significant differences were found with regards to the AO fracture type comparing the two groups. Type 44B3 injuries were significantly more likely to be insufficiently reduced in the cast (84.2% versus 62.3%; *p* = 0.004). (Table 2).

**Table 2:** Fracture classification according to AO stratified by the quality of initial closed reduction

	All patients (*n* = 134)	Sufficient reduction (*n* = 77)	Insufficient reduction (*n* = 57)	*p*
AO classification, N (%)				
44B1	10 (7.5)	6 (7.8)	4 (7.0)	1.000
44B2	14 (10.4)	13 (16.9)	1 (1.8)	0.598
44B3	96 (71.6)	48 (62.3)	48 (84.2)	**0.007**
44C1	4 (3.9)	3 (3.9)	1 (1.8)	0.636
44C2	10 (7.5)	7 (9.1)	3 (5.3)	1.000

Initial ankle dislocation was significantly less severe in the group with sufficient reduction versus the group with insufficient reduction (medial clear space 10.1 versus 14.6 mm; *p* = 0.001). In addition, a significant difference was found with regard to the percentage of initial dislocation (26.0 versus 32.3%; *p* = 0.045).

Posterior malleolar fracture was present in 106 patients (79.1%) and was not significantly different in patients with insufficient reduction and sufficient reduction (76.6% versus 82.5%, *p* = 0.520). There were no significant differences in the posterior malleolar fragment between the patients with insufficient reduction and those with sufficient reduction, such as in the sagittal diameter (17.0 mm versus 18.0 mm; *p* = 0.569) or percentage of the tibial articular surface (15.8 versus 16.3%; *p* = 0.805). (Fig. 1) (Table 3). There was no difference with regards to the Haraguchi classification of the PMF comparing the two groups.

**Table 3:** Comparison of the configuration of the PMF and the initial dislocation in patients with sufficient versus insufficient reduction during splint immobilization

	All patients (*n* = 134)	Sufficient reduction (*n* = 77)	Insufficient reduction (*n* = 57)	*p*
Initial dislocation (medial clearspace), mm, mean±SD	12.0±5.4	10.1±4.45	14.6±5.52	**0.001**
Percentage of initial dislocation, %, mean±SD	28.7±20.8	26.0±21.5	32.3±19.3	**0.045**
Presence of PMF, N (%)	106 (79.1)	59 (76.6)	47 (82.5)	0.520
Haraguchi classification				
1	50 (37.3)	26 (44.2)	24 (50.0)	0.202
2	12 (9.0)	7 (9.6)	5 (13.0)	0.202
3	44 (32.8)	23 (46.2)	21 (37.0)	0.202
Percentage PMF in tibial joint surface, %, mean±SD	16.0±11.1	15.8±11.2	16.3±10.0	0.805
Sagittal diameter of PMF, mm, mean±SD	17.5±9.0	17.0±7.95	18.0±10.0	0.569

Bivariate analysis was performed to identify risk factors for insufficient reduction. Stepwise logistic regression analysis identified initial medial clearspace at the time of dislocation as risk factor independently associated with insufficient reduction (standardized Beta (95% CI): 0.432 (0.024–0.058); adj. *p* = 0.001). The percentage of PMF did not proof to be a risk factor for insufficient reduction (standardized Beta (95% CI): -0.034 (-0.502-0.340); adj. *p* = 0.704).

**Fig. 4:**
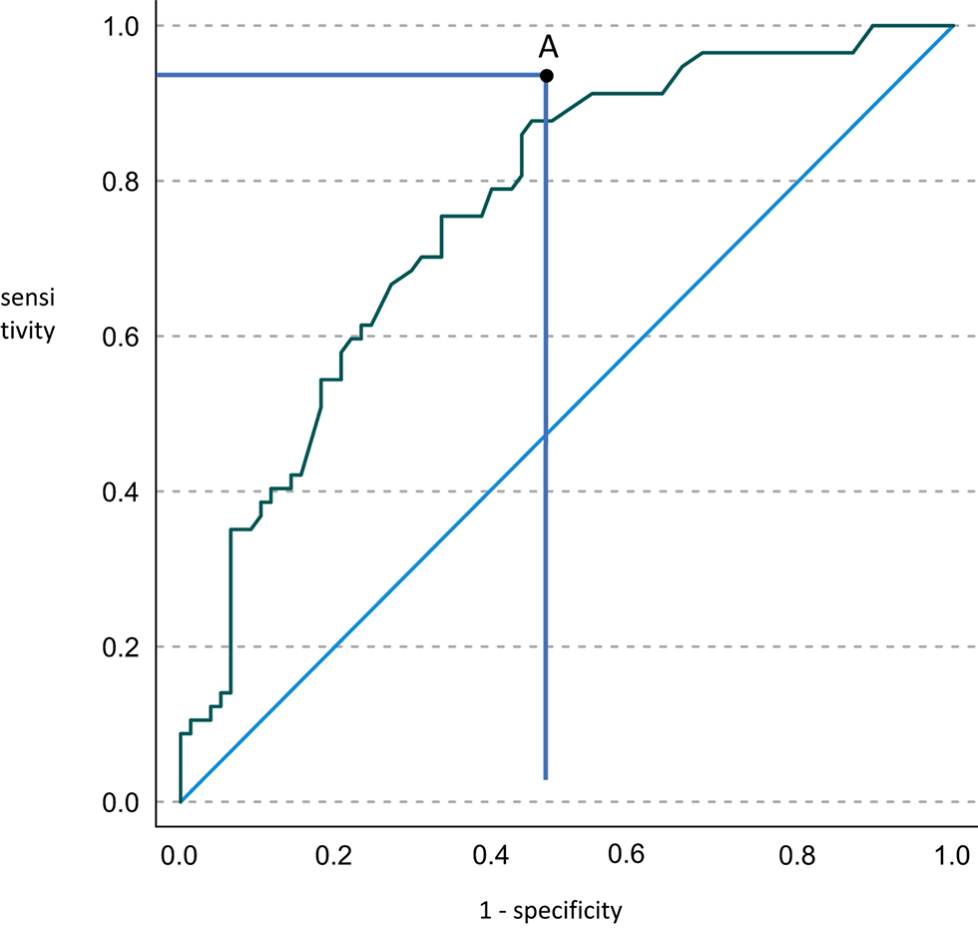
ROC curve analysis for initial medial clearspace to predict insufficient reduction. Point A indicates a medial clearspace of 9 mm

The ROC-analysis (Fig. 4) of the initial medial clearspace resulted in an area under the curve of 0.763 (95% CI, 0.682–0.843; *p* < 0.001) with an overall model quality of 0.68. An initial medial clearspace at the time of dislocation of 9 mm demonstrated a sensitivity of 88% and a specificity of 55% for insufficient reduction.

The evaluation of the time to final surgery showed a shorter waiting time in the insufficient reduction group with a mean of 4.65 ± 3.5 (range 4–31 days) compared to 6.0 ± 5.0 days (range 2–18 days) (*p* = 0.036). The time from accident to discharge also differed significantly between the two groups. The group with insufficient reduction showed a significantly shorter hospitalization time (10.8 ± 5.6 days in the group with insufficient reduction compared to 14.2 ± 10.7 days in the group with sufficient reduction, *p* = 0.030). There were no differences in operation time between the two groups (insufficient reduction 127 ± 45.5 min (range 60–232 min) versus 120 ± 50.8 min (range 45–441 min) (*p* = 0.393)).

## Discussion

In our study group, in half of the patients with a dislocated ankle fracture a sufficient reduction and retention with immobilization in a cast was achieved. However, in 42.5% of patients, the reduction after closed reduction and cast application was found to be insufficient in the first xray.

Wawrose et al. contributed a study and compared the temporary ankle spanning external fixator application (*n* = 28) with casting (*n* = 28) following ankle fracture dislocation. The authors showed a 50% loss of reduction in the casting group, but did not define the loss of reduction and did not evaluate possible causes of loss of reduction in more detail [15]. Other studies found the type of cast to be a significant risk factor for a loss of reduction. Baker et al. showed a 50% rate of loss of reduction in a plaster cast versus 0% loss of reduction in a bivalve fiber glass cast group [19]. 

The results of the present study show that fractures found to be insufficiently reduced during cast immobilization initially had a significantly higher medial clearspace at the time of dislocation, and percentage of dislocation was significantly higher compared to the sufficient reduced fractures. In addition, the initial medial clearspace during dislocation proved to be an independent risk factor for insufficient reduction. The size or presence of the PMF was found to have no influence in our study.

Previous literature has shown that the size of the PMF and the presence of the PMF are often a risk factor for redislocation. Gerlach et al. compared the temporizing immobilization in a cast versus an external fixator and could show that the PMF-size was an important predictor of loss of reduction and recommended therefore an external fixator in those with a PMF-size > 22% of the tibial joint surface [25]. In the study by Matson et al., a greater PMF fragment size was associated with higher rates of interim failure of closed reduction in closed ankle fracture-dislocations. The authors found a rate of failure of 65% when the ratio of PMF fragment size to complete articular surface was > 0.1 compared to 18% when the ratio was ≤ 0.1 (*p* = 0.016) [16]. 

In contrast, however, in a more recent study by Mandelka et al. the presence of a PMF had no influence on the loss of reduction [22]. 

One reason why the medial clearspace acts as an independent risk factor for an insufficient reduction could be that a dislocation with a greatly increased medial clearspace leads to a tearing of all ligamentary structures and thus to increased instability during cast immobilization.

It must also be discussed that there could be a selection bias, as the fractures that could not be reduced under the xray and directly received an external fixator were not included in our study.

Demographic factors and comorbidities did not differ significantly between the two groups in the present study. This is in concordance with previous studies, showing that age, gender, and injury mechanism (supination versus pronation) are not associated with the ability to achieve reduction acutely [19, 29]. 

In our study, those patients who were insufficiently reduced in the cast had a significantly shorter time to final surgery and a shorter time between accident and discharge. This actually contradicts the results of the studies which showed that poor reduction leads to prolonged swelling and prolonged hospitalization [19, 20]. However, one reason could be that patients who were known to have insufficient reduction in plaster were operated on more proactively.

The present study has several limitations. First, this study is retrospective in nature and thus is susceptible to bias from limited available information. Furthermore, all radiographic measurements were performed by a single investigator, with the potential risk of a measurement bias. Second, there was heterogeneity in the group of physicians performing the initial reduction and cast application. While this may have led to some inconsistency with clinical techniques, it may also render the data more generalizable for the larger population of ankle fracture-dislocations and more specifically to other providers at different acute treatment centers. In addition, some patients were reduced under sedation, if possible due to pain, and others under short anesthesia. This can also lead to a bias in the results. Finally, it is well-known that insufficient reduction may result in significant complications for the patients (such as soft tissue problems or prolonged swelling), but the exact definition of insufficient reduction is unclear. The definition for insufficient reduction used in this study was based on the principle that a medial clearspace > 4 mm is considered pathologic in the literature [30, 31]. Murphy et al. likewise showed that the medial clearspace can vary up to 7 mm depending on the type of radiograph. We therefore set the cut-off value at 5 mm [32]. 

## Conclusion

An increased initial medial clearspace proofed to be an independent risk factor for insufficient reduction in a cast.

We therefore recommend primary external fixation or acute internal fixation, if the soft tissue allows it, for those patients with increased initial medial clearspace at the time of the dislocation.

## Data Availability

No datasets were generated or analysed during the current study.

## Abbreviations

AO/OTA: AO Foundation/Orthopedic Trauma Association
BMI: Body Mass Index
ORIF: Open reduction internal fixation
PMF: Posterior malleolar fragment/Volkmann fragment
ROC: Receiver operating characteristic
SD: Standard deviation
